# Machine Learning Application in Horticulture and Prospects for Predicting Fresh Produce Losses and Waste: A Review

**DOI:** 10.3390/plants13091200

**Published:** 2024-04-25

**Authors:** Ikechukwu Kingsley Opara, Umezuruike Linus Opara, Jude A. Okolie, Olaniyi Amos Fawole

**Affiliations:** 1 SARChI Postharvest Technology Research Laboratory, Africa Institute for Postharvest Technology, Faculty of AgriSciences, Stellenbosch University, Stellenbosch 7600, South Africa; ikekings101@gmail.com (I.K.O.); opara@sun.ac.za (U.L.O.); 2 Department of Food Science, Stellenbosch University, Stellenbosch 7600, South Africa; 3 UNESCO International Centre for Biotechnology, Nsukka 410001, Enugu State, Nigeria; 4 Gallogly College of Engineering, University of Oklahoma, Norman, OK 73019, USA; jude.okolie@ou.edu; 5 Postharvest and Agroprocessing Research Centre, Department of Botany and Plant Biotechnology, University of Johannesburg, Johannesburg 2006, South Africa

**Keywords:** machine learning, models, prediction, forecast, postharvest, losses and waste, fruit, vegetables, horticulture, quantification

## Abstract

The current review examines the state of knowledge and research on machine learning (ML) applications in horticultural production and the potential for predicting fresh produce losses and waste. Recently, ML has been increasingly applied in horticulture for efficient and accurate operations. Given the health benefits of fresh produce and the need for food and nutrition security, efficient horticultural production and postharvest management are important. This review aims to assess the application of ML in preharvest and postharvest horticulture and the potential of ML in reducing postharvest losses and waste by predicting their magnitude, which is crucial for management practices and policymaking in loss and waste reduction. The review starts by assessing the application of ML in preharvest horticulture. It then presents the application of ML in postharvest handling and processing, and lastly, the prospects for its application in postharvest loss and waste quantification. The findings revealed that several ML algorithms perform satisfactorily in classification and prediction tasks. Based on that, there is a need to further investigate the suitability of more models or a combination of models with a higher potential for classification and prediction. Overall, the review suggested possible future directions for research related to the application of ML in postharvest losses and waste quantification.

## 1. Introduction

Horticultural produce is known to contain essential nutritious elements in large quantities [1,2,3]. These essential nutrients are vital to maintaining a healthy life and have many benefits for the human body [4]. Chronic diseases such as hypertension, heart disease, stroke, diabetes, cancer, and pulmonary disease are the leading causes of mortality [5]. Increasing cases of obesity and malnutrition are also a growing concern worldwide. Research evidence has shown that increased fruit and vegetable consumption decreases the risk of diseases [6]. Also, there is a correlation between fruit and vegetable consumption and delays in age-related disorders [6,7]. Despite the benefits obtained from the consumption of fruit and vegetables, a remarkable amount is still wasted globally throughout the food value chain for several reasons, such as pest and disease infestation, environmental stress, quality issues, and marketing aesthetic standards [8,9]. To address these challenges, artificial intelligence (AI), particularly ML, has emerged as a promising tool in preharvest and postharvest horticulture [10].

**Figure 1:**
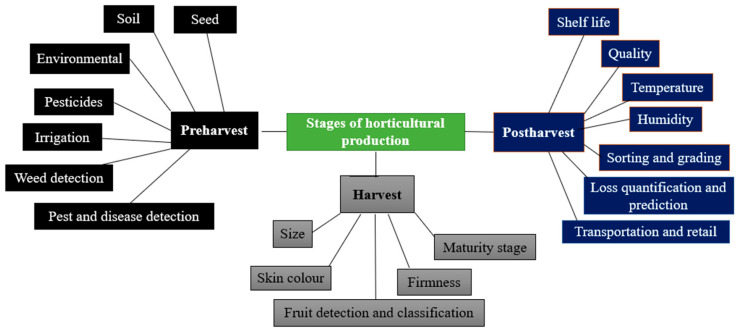
Stages of the horticultural value chain where ML can be applied. Adapted with a slight modification from [11].

Horticulture is critical to supplying fruit and vegetables, which are rich in essential nutrients and contribute significantly to global economies. Despite their importance, horticultural practices in various regions remain rudimentary, often due to barriers such as a lack of modern technology. This is because these farmers are reluctant to use modern technologies for several reasons, such as a lack of skill and the cost of acquiring such technology [11]. This results in high preharvest and postharvest losses. Deficiencies in data-driven decision-making related to weather, soil conditions, irrigation, and pest management contribute to inefficiencies that can be addressed through ML technologies. The integration of ML algorithms into the horticultural value chain (Figure 1) can enhance produce quality and optimize resource allocation, thereby increasing the return on investment for stakeholders [12].

Recent technologies in machine vision, sensors, and remote satellite data generation have produced big data at different stages of the food value chain. As a result, the advent of big data technologies has catalyzed the application of ML in various stages of the food value chain.

ML has been widely used in different fields of agriculture and plant science, such as plant breeding [13], in vitro culture [14], stress phenotyping [15], stress physiology [16], plant system biology [17], plant identification [18], plant genetic engineering [19], and pathogen identification [20]. Despite the growing body of research on ML in agriculture, there is a noticeable gap in the literature concerning its application in postharvest loss and waste management. The available literature is mainly focused on the application of ML technologies in preharvest horticulture and on the classification of fruit and vegetables for sorting and grading [21,22,23,24,25]. A Scopus search with filters for the past decade showed 89 reviews for ML combined with deep learning, AI, and machine vision applications in agriculture and food production. A further investigation shows that only seven reviews included information on the application of ML in postharvest horticulture [26,27,28,29,30,31,32]. Given this gap, the present review aims to synthesize the current knowledge on ML applications in preharvest horticulture and extend the discussion to postharvest scenarios, including processing and retail. The review structure is as follows: firstly, an overview of the concept of the ML technique in horticultural production was presented, followed by the application of ML in preharvest horticulture. Further discussion was presented on the application of ML in postharvest handling and processing. In addition, the application of ML in retail was discussed. Lastly, the prospects of the application of ML in postharvest loss and waste quantification and prediction and prospects in the area were discussed.

### 1.1. The Concept of ML Technique—An Overview

ML is a subset of AI focused on the development of algorithms and statistical models that enable computers to learn and make predictions or decisions without being explicitly programmed for specific tasks. While AI is a broader concept that encompasses machines designed to mimic human intelligence, including reasoning, learning, and problem-solving, ML specifically deals with the learning aspect, where machines improve their performance on a task through exposure to data. In essence, all machine learning is AI, but not all AI is machine learning; AI includes rule-based systems, expert systems, and other methods that do not necessarily involve learning from data.

In the current era, vast amounts of data are generated across various domains, presenting opportunities and challenges for data analysis. These data can be used for validation, calibration, classification, verification, prediction, and characterization of variables. However, using manual approaches to process and perform tasks with the generated data has resulted in several challenges due to the size and complexity of the data [33]. To resolve this problem, a sub-field of AI known as ML emerged to automatically learn and capture the relationship between various features in data to produce a result used in decision-making. The primary aim of ML is to formulate algorithms capable of autonomously discerning patterns in data and making informed predictions or decisions [34]. The process involves feeding ML models with a large amount of data and allowing them to learn the features and patterns within the data.

ML has gained significant attention in recent years due to the increasing availability of big data in different fields and the need to harness these data to solve problems [35]. ML has been applied in various fields, such as transportation, telecommunication, healthcare, finance, and agriculture. In the agricultural sector, ML has found applications in areas such as crop yield prediction [36,37], pest and disease detection [38], and cultivar classification [39]. By leveraging ML, farmers can optimize their production inputs and improve their yields, thereby enhancing food security.

Although ML was initially conceived as a complement to traditional statistical methods, it offers distinct advantages, such as automation and the capacity for self-improvement through iterative learning [40]. As defined by Wang et al. [40], ML deals with the development of intelligent models that use algorithms to predict, estimate, and classify a variable. The performance of an ML model is contingent upon the quality of feature analysis, data preprocessing techniques, and the efficacy of the algorithmic methods employed [40].

### 1.2. Machine Learning Models

**Figure 2:**
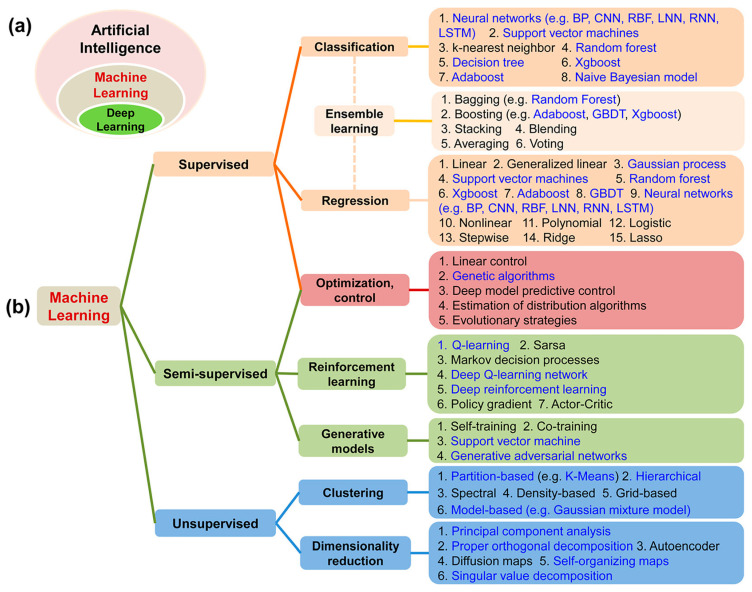
Types of ML models. Adapted from [42]. (**a**) Relationship between AI, ML, and deep learning (DL); (**b**) Classification of ML algorithms based on learning technique.

There are different types of learning in ML models—supervised, semi-supervised, unsupervised, and reinforced learning (Figure 2). The main difference between supervised and other learning types is that the datasets used are labeled with features to known outputs. In semi-supervised learning, the datasets used are both labeled and unlabeled, and usually, the numbers of unlabeled data are higher than the labeled data [41]. Unlike supervised learning, unsupervised learning uses unlabeled datasets to learn patterns and predict output. Reinforced learning allows the model to simulate its environment and make predictions based on the state of the environment.

ML models like random forest (RF), K-nearest neighbors (KNNs), and linear regression (LR) are designed to identify and learn patterns within datasets [43]. The predictive accuracy of a machine learning model generally improves with increased exposure to relevant data [44]. Models are widely used for predictive analysis as data features are learned and tuned to improve performance. ML models can be broadly categorized into supervised, semi-supervised and unsupervised learning algorithms as shown in Figure 2. Supervised learning algorithms are trained on labeled datasets, allowing them to make predictions or classifications, whereas unsupervised learning algorithms work with unlabeled data to identify underlying patterns or structures [45]. In horticultural applications, supervised models like RF and KNN have been effectively used for tasks such as disease detection and yield prediction, while unsupervised models find utility in clustering similar types of produce based on features like size, color, and texture. Other advanced models like SVM and neural networks are also gaining traction in horticultural applications, offering higher accuracy and the ability to model complex relationships in data [37].

**Figure 3:**
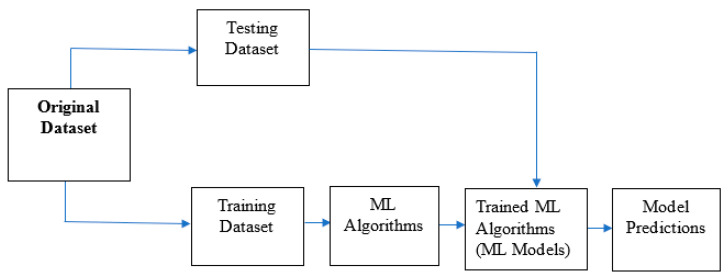
A basic model of ML, adapted from [45].

The architecture of learning and prediction activities in ML is presented in Figure 3. The original data are usually split into two—a training dataset and a test dataset. In some cases, there is a third dataset for model validation after testing. The training dataset is used to train the model, while the test dataset is used to evaluate the performance of the model. The model makes predictions using the observed pattern in the training dataset without knowing the true target variable. The predictions of the model are then compared to the true target variables, and performance parameters are recorded [40].

### 1.3. Parameters Used to Evaluate the Performance of an ML Model

The evaluation of an ML model’s performance relies on a set of metrics that quantify its accuracy and reliability. According to Raschka [46], the parameter for evaluating an ML model is mostly the all-around performance of the model. This is important in identifying the ideal model to perform a task. Commonly employed metrics for assessing model performance include the confusion matrix, classification accuracy, cross-validation, F1-score, precision, and recall [11]. In horticultural applications, metrics like precision and recall are particularly important when the cost of false positives and false negatives, such as misclassifying produce quality, can be high.

## 2. ML Application in Preharvest Horticulture

While ML has extensive applications in diverse fields like telecommunication and healthcare, its utility in preharvest horticulture is increasingly recognized [40]. Recent studies have highlighted the growing role of ML in preharvest horticulture, particularly in areas like disease and weed detection, yield prediction, and crop quality assessment [43,47,48].

### 2.1. Pest and Disease Prediction and Detection

In horticulture, the timely detection of pests and diseases is crucial for implementing effective control measures. This is because it enables farmers to plan mitigation and control measures [49]. Disease detection can be time-consuming, especially where traditional laboratory methods are used [50]. The methods often come with limitations, such as high costs and time consumption [50]. As a result, AI-based techniques that use cameras for image acquisition have recently emerged for disease detection. Emerging AI-based techniques leverage ML and deep learning algorithms to enhance pest and disease recognition accuracy and speed. These technologies allow for early detection and management of pests and diseases to increase yield [51]. This is important because pest and disease infestations affect the quality of horticultural products and may lead to losses or waste [49].

A growing body of research has focused on employing ML algorithms to detect and predict pests and diseases in horticulture (Table 1). Pantazi et al. [21] investigated the infection of milk thistle by smut fungus. Three ML models—supervised Kohonen network, counter propagation artificial neural network, and XY-fusion network—were used to identify infected and healthy plants during plant growth. In a similar study, Chung et al. [52] applied the support vector machine (SVM) to classify rice seedlings infected by Bakanae disease from the healthy ones. Maniyath et al. [50] applied several ML models to distinguish between healthy and disease-infected papaya leaves. The authors reported that the random forest (RF) model outperformed other models with an accuracy of 70.14%. Kasinathan et al. [51] classified and detected insects in field crops using artificial neural networks (ANNs), SVM, KNN, naïve Bayes (NB), and convolutional neural network (CNN) models. The study was based on the shape features of the insect, and the results showed that the CNN is a suitable classification model for the study in comparison with the other models. Skawsang et al. [49], in another study, applied ML models to forecast the occurrence of pests using metrological and plant phenology data. The study aimed to provide an early warning system for effective pest control. In another study, Javidan et al. [53] developed a method to classify disease-infected and healthy grape leaves. The authors used principal component analysis (PCA) to reduce the data dimension before subjecting the data to SVM classification. The result showed that the SVM classifier combined with the linear kernel, using the gray-level co-occurrence matrix (GLCM) features, produced a 98.71% accuracy. Mohammed et al. [54] developed a method of predicting date palm mite infestation on date fruits using meteorological variables and the physicochemical properties of date fruits integrated into LR and decision forest regression (DFR) models. The authors reported that when the meteorological and physicochemical properties were combined, the model was able to predict the date palm mite count on date palm fruits with an accuracy of R^2^ = 0.918. Collectively, these studies suggest the potential of ML algorithms to provide accurate and timely predictions, aiding in the development of effective management strategies for pest and disease control. This is important in planning management strategies to avoid fruit and vegetable losses and waste due to pest and disease defects and damage.

### 2.2. Prediction and Detection of Crop Loss Due to Natural Causes

Extreme weather and climatic conditions pose significant risks for on-farm crop loss, often beyond the control of standard farming operations [57]. Farmers often rely on agricultural insurance as a financial safety net to mitigate these risks. Insurance institutions perform field evaluations to estimate the yield loss in the event of crop loss through a natural disaster. The accuracy of insurance estimations often hinges on identifying “Homogeneous Damage Zones”, which facilitate the extrapolation of localized data to estimate losses across an entire field [57].

Table 2 summarizes various ML applications designed to detect and predict crop loss arising from natural causes. To investigate damaged zones in fields affected by hailstorms, Sosa et al. [57] developed a method that combined sentinel images with damage evaluation data to determine damaged zones in fields affected by hailstorms to help in insurance claims. In a similar study, Li et al. [58] developed a system to investigate drought risk and its effect on wheat production in the North China Plain. The study also aimed to guide agricultural insurance, which could be a serious issue when a natural disaster causes damage to a farm. The authors fed the result of the model to simulate the crop–weather relationship over a large area (MCWLA) into an RF and multiple linear regression (MLR) models to estimate losses due to drought in three different scenarios—mild drought, moderate drought, and severe drought. In another study, Xu et al. [59] applied the SVM model since it performed better than the ANN to predict the distribution of frost damage to tea trees in the Zhejiang Province of China in 2016/2017. The authors used meteorological data to predict the future occurrence of frost events to help farmers in their decision-making. In a recent study, Prodhan et al. [22] estimated future drought and its effect on yield loss in South Asia using an ensemble ML (EML) that was embedded with RF and a gradient boosting machine (GBM). The model performed optimally in predicting yield loss risk for rice, wheat, and maize crops, with a root mean square error (RMSE) lower than that of RF and GBM as stand-alone models.

The existing literature Indicates limited studies focused on predicting crop loss due to natural causes, often attributed to the limited availability of comprehensive data. This can be attributed to the limited available data [58]. The accurate prediction of crop loss due to natural causes in a wide area mostly relies on historical events as input data, but detailed information on damaged areas and losses is mostly unavailable. Given these challenges, future research should prioritize the characterization of natural events affecting crop production, as accurate predictions are crucial for global food security.

### 2.3. Yield Prediction

Achieving optimum yield in horticultural production is critical, as it directly impacts the farmer’s return on investment and broader food security. Within the framework of precision agriculture, accurate yield prediction is indispensable for enabling proactive planning and decision-making by farmers and other stakeholders in the value chain [63]. Also, yield prediction is essential for matching demand with supply. Lastly, yield prediction is fundamental to helping farmers know the right harvest time to avoid storage loss [10]. However, yield prediction is complicated by various factors, including weather conditions, soil properties, and pest incidence, which introduce significant variability. Given these complexities, developing automated yield prediction systems using machine learning algorithms is increasingly seen as a necessity.

Recent studies have applied different ML models to predict crop yield in different scenarios. Ramos et al. [64] used a machine vision system and an image-processing model to detect and classify fruit. The system developed in the study was used to count coffee fruit on the tree branches and classify the fruit as harvestable or not harvestable. In another study, Sengupta and Lee [65] applied the SVM to identify the number of immature green citrus fruit in a tree canopy. The result showed that the model accurately identified and counted 80.4% of the fruit. Abbas et al. [66] developed a model to predict the yield of potatoes in the Atlantic Region of Canada using LR, elastic net (EN), KNN, and support vector regression (SVR). The result of the study was important for establishing field-specific management practices for potato growers in the area. Similarly, to predict the yield of Irish potatoes and maize in the Musanze district in Rwanda, Kuradusenge et al. [37] applied the RF to determine the effect of temperature and rainfall on crop yield. The study aimed to provide farmers with early information on the expected climatic conditions to mitigate climate change’s impact on crop production. In a similar study, Iniyan et al. [36] used several models (LR, decision tree (DT), elastic net, Lasso regression, Ridge regression, partial least square regression (PLSR), gradient boost regression (GBR), and long short-term memory (LSTM)) to predict yield loss based on historical agronomical data gathered in 18 years. The agronomical data used in the study have more variables (temperature, precipitation, humidity, soil type, crop type, season, and field area) than most of the published literature, which improves the reliability of the results of the models. Khan et al. [67] describe how plant height, fruit production, slope, leaf loss, and blower damage can be used to predict yield loss during the mechanical harvesting of wild blueberry. The authors applied SVR, LR, and RF to predict losses, and the study’s overall result could help optimize the harvesting technique for loss reduction. These studies demonstrated the efficacy of diverse ML algorithms in yield prediction, providing valuable insights for farmer decision-making and planning. Compared to other non-regression ML models, regression models such as SVR, LR, and RF have several advantages for prediction, hence their application for yield, pest and disease, and crop loss prediction in the studies. Regression models are used to investigate vital relationships between targeted variables of interest and the predictor variables [68] due to their ability to form associations between dependent and independent variables. Additionally, these models allow prediction through time series data and show the underlying relationship among variables [69]. For instance, an LR model easily fits a single parameter (predicted output) and captures a nonlinear relationship between predictor and response variables. This straightforward attribute of LR makes it the most used model for prediction tasks [69]. Table 3 summarizes the key studies that have employed ML models for yield prediction.

### 2.4. Crop Quality

The quality assessment of horticultural produce is critical for determining compliance with market standards, thereby influencing marketability and pricing. Therefore, accurate quality classification is pivotal for aligning produce with market standards, optimizing pricing, and minimizing postharvest losses and waste [48,73]. Factors such as temperature, humidity, farming method, and packaging affect preharvest and postharvest crop quality [10]; other factors could be contamination due to foreign materials [74]. These factors could result in economic loss because of postharvest losses and waste, with a broader implication for natural resources used for food production and the environment.

ML technologies offer a time-efficient and highly accurate approach to quality classification in horticultural produce. Zhang et al. [74] applied linear discriminant analysis (LDA) and SVM to classify foreign material inside cotton lint. The study reported a 95% accuracy in the classification of cotton lint by the SVM model. Zulkifli et al. [75] developed a model that combined a machine vision system with discriminant analysis and the SVM model to predict the ripening stages of papaya. The model performed optimally, with LDA producing the highest result accuracy of 83.5%. In another study, Agarwal et al. [76], designed an SVM, KNN, multi-layer perceptron (MLP), and NB method to classify wheat grains into ‘fresh’ and ‘rotten’. The authors reported that SVM produced the highest accuracy of 93% based on color features, while the NB model produced the highest accuracy of 65% based on texture features. The result proved SVM to be a strong discriminatory model as it tended to classify with the highest accuracy with color features, which are regarded as possessing high discriminatory features in comparison to texture features [76]. Occhiuzzi et al. [77], developed an RFID-based system that was aimed at controlling the environmental conditions of stored avocado fruits and detecting ripening status. The system fed the data retrieved from the tag reader into an SVM that classified the fruits into “unripe”, “stock”, “grocery”, and “consumer” with more than 85% accuracy. Researchers have prominently used SVM for classification tasks and compared its performance to other classifiers [76,78,79]. Their results demonstrated SVM’s ability to classify variables with good accuracy. This is due to its generalization ability, robustness, and simple principle, which make it arguably the most popular model for supervised learning [80]. The industrial-scale adoption of these machine learning technologies holds significant promise for reducing postharvest losses attributable to suboptimal produce quality. Table 4 summarizes key studies employing machine learning models for quality assessment in horticultural produce.

## 3. ML Application in Postharvest Handling and Processing

The recent literature indicates a growing application of machine learning technologies in postharvest handling and processing, particularly in fresh produce sorting, grading, and cultivar classification. These two postharvest activities are traditionally manual and subjective and are based on physical attributes such as shape, color, and the presence of blemishes [85,86]. The labor-intensive and time-consuming nature of traditional manual methods, coupled with their subjectivity, has led to the adoption of machine learning technologies to address these challenges.

### 3.1. Fruit and Vegetable Sorting/Grading

The sorting of fresh produce is a quality classification activity and is greatly affected by the market standard, especially for export commodities [9]. According to Opara et al. [9], sorting fruits and vegetables is important because aesthetics is a significant attribute in fresh produce grading, determining the quality and monetary value of such fresh produce. Many traditional sorting processes involve an individual’s physical identification of fresh produce based on specific attributes that are sometimes straining, time-consuming, and dependent on the sorter’s perception [85,86,87,88]. Also, traditional sorting methods are prone to inaccuracy due to fatigue and lack of training [89], and this may contribute to losses [87,90]. Adopting ML technologies is thus crucial for enhancing efficiency and productivity in sorting and grading fresh produce.

Table 5 summarizes key studies focused on enhancing the efficiency of sorting and grading systems in postharvest horticultural production through ML technologies. Caladcad et al. [23] developed an acoustic signal system to classify coconut fruit into three groups—pre-mature, mature, and over-mature. The data from the study were subjected to ANN, RF, and SVM models, and the results showed that the RF model outperformed others with 83.48% accuracy. This classification system can significantly benefit the large-scale processing of coconut fruit for mass exportation. Ai et al. [91] applied the RF model to discriminate between premium quality oil (extra virgin olive oil) and inexpensive edible oils. The study was based on the fatty acid methyl esters of the oils. The authors aimed to find a close substitute, a cheap oil with similar nutritional content to the expensive extra virgin olive oil. As with many related studies, Piedad et al. [87] developed a model to sort bananas by tiers rather than by individual fruit. The study classified banana tiers into four classes—extra class, class I, class II, and reject class—using color and size features. In another study, Ireri et al. [85] reported a machine vision system that used color images and the radial basis function–support vector machine (RBF-SVM) classifier to detect healthy tomatoes and those with defects. The study aimed to develop a low-cost grading system to grade tomatoes on the processing line. The system successfully classified tomatoes into four categories using color, texture, shape, and combined features. A recent study by Bhargava et al. [92] proposed an automated system to detect fruit and vegetable types and grade them using various features such as color, texture, and geometrical features. The system utilized LR, the sparse representative classifier (SRC), ANN, and SVM, with SVM producing the highest accuracy for both fruit and vegetable detection and grading. Fruit and vegetable grading and sorting is a classification task mostly performed by classification models (Table 5). The results of the studies depict that several algorithms perform satisfactorily for classification due to the high accuracy achieved in the studies. However, SVM is a binary classifier that performs by finding the best subspace that optimally separates variables into classes [76] and has a high computational efficiency and generalization capability [80]. Therefore, the combination of SVM and other models would have a potential for higher accuracy for prediction and classification. SVM is also known for reduced computational time and the ability to use the kernel trick to delineate data into a higher-dimensional space before actual classification [80].

These studies demonstrate the potential for scaling up ML systems in industrial settings to mitigate the challenges associated with traditional manual methods in fresh produce sorting and grading.

### 3.2. Crop Detection and Cultivar Classification

ML models have been increasingly employed to detect and recognize various crop types [97,98,99]. Similar technology has also been employed to differentiate fruit and vegetable cultivars according to the specific market and industrial needs [98,99,100,101]. Accurate classification is crucial for meeting market specifications, thereby minimizing the risk of rejection and subsequent loss or waste. In their study, Filho et al. [97] developed a methodology and model to detect and map rice crops in the field from the Sentinel-1 time series using deep learning (LSTM and bidirectional LSTM (Bi-LSTM)) models. The performance of deep learning models like LSTM and Bi-LSTM was compared against traditional machine learning models, including SVM, RF, KNN, and NB, to evaluate their efficacy in crop classification. The ML models achieved high accuracy in classifying rice as the LSTM. ML in cultivar classification was reported by Hu et al. [98]. The authors differentiated the Korla fragrant pear into two—deciduous-calyx pear and persistent-calyx pear—using successive projection algorithms and SVM to establish classification, with SVM achieving an accuracy of 96.7%. Yang et al. [99] applied DT, KNN, naïve Bayes (NB), linear discriminant analysis (LDA), SVM, and back propagation neural network (BPNN) to classify apricots based on their shape features. The study aimed to develop a model for cultivar classification of apricot fruit using shape features to distinguish the different cultivars. In a similar study, Khatri et al. [101] applied KNN, NB, classification and regression tree (CART), and ensemble methods (EMs) to distinguish wheat seeds into three varieties: Kama, Rosa, and Canadian. Using the physical features of the seeds, the authors reported that the EM produced the highest result with 95% accuracy. Table 6 summarizes key studies that have applied machine learning technologies for crop detection and cultivar classification.

## 4. ML Applications during Retail

In retail, sustainable decision-making is crucial for long-term viability and competitive advantage. This means that decisions are made to enhance profitability, return on investment, and minimize risk. To make these decisions, forecasts are made using the past and present trends of activities around the business [108]. Many factors influence retail operations, including market dynamics and consumer behavior, making accurate forecasting a challenging yet essential task. ML applications have been deployed to provide techniques to simulate, detect, and predict aspects of the complex retail system for timely decision-making for efficient operation and to reduce food waste generation.

Some studies have applied ML techniques to enhance retail operations. For instance, as indicated in Table 7, Myat and Tun [109] used the RF classification model to predict palm oil prices in Myanmar using data obtained from the Myanmar Edible Oil Dealers Association (MEODA). The prediction was conducted to determine whether the price will rise so that imported palm oil can be traded in the local markets. In another study, Valecha et al. [110] used the RF classifier to classify customer behavior to buy products based on attributes such as interpersonal, individual, environmental, and organizational factors based on the shopping pattern (Table 7). Customer behavior prediction was based on data collected from the Kaggle repository, and the study showed 94% accuracy. To predict future sales, Dairu and Shilong [111] proposed a technique developed by applying the eXtreme gradient boosting (XGBoost) model to forecast sales by extracting features from historical sales data. The study found that the XGBoost model yielded superior performance, achieving an RMSE of 0.878, thereby outperforming the LSTM and ARIMA models. In a similar study, Swami et al. [112] predicted the total product and store sales using XGBoost, LSTM, and autoregressive integrated moving average (ARIMA) models. The result revealed that the XGBoost outperformed the other models with an RMSE of 0.878. The authors reported that XGBoost is mainly used in Kaggle competitions and efficiently handles different sparsity patterns.

## 5. ML Application in Postharvest Loss and Waste Quantification of Fresh Horticultural Produce

Due to their high moisture content and limited shelf life, fruits and vegetables are particularly vulnerable to postharvest losses. Globally, fruit and vegetables account for the highest food commodities lost or wasted. According to the FAO, wastage is as high as 37–55% [117]. The successful application of ML for yield prediction [64], disease detection [21], and crop quality evaluation [74] suggests that the application of ML to quantify and predict postharvest wastage along the food value chain would prove fundamental in production planning and policymaking. Despite the apparent use of ML in this domain, there is a noticeable lack of research focusing on its application for quantifying physical postharvest losses in fresh produce. Yu et al. [118] used factors such as family status, income, expenditure, and grain transaction and applied the bias classifier, DT, and SVM models to predict grain losses. The classification result was compared among the three models, and SVM achieved the highest result with an accuracy of 97.30%. A literature search suggests a few studies similar to Yu et al. [118] that used socio-economic factors to classify food loss and mostly used grains. These studies did not include actual physical quantification of the postharvest losses but rather focused on the causes and classification of the problem using secondary data.

Several factors contribute to postharvest losses along the food value chain [8,119,120]. Some of the factors are value chain-specific, while others occur throughout the value chain. As a result, to effectively quantify postharvest losses using ML, different types of data are needed, as determined by the hotspot being assessed. The type of data could include data on environmental conditions (such as temperature and relative humidity), data from storage methods (such as freezing and drying), and data during transportation and logistics (such as the rate of impact, vibration, and compression). Also, data collected during physical loss quantification by weighing or counting is believed to be highly accurate and could be used for ML quantification of postharvest losses.

Given the gap in the application of ML for physical postharvest loss and waste quantification, there is an urgent need for research that employs ML techniques to quantify postharvest losses of horticultural produce using physically quantified data. Such an approach would be invaluable for evidence-based policymaking and implementation.

## 6. Limitations of Implementing ML Techniques in Horticultural Production and Future Prospects

Although ML techniques improve horticultural production through automation and enhanced efficiency, there are several challenges and limitations associated with their implementation. These challenges arise due to the complexities of horticultural production. One of the major challenges is data availability and variability. Data for horticultural studies come from several sources, such as laboratory experiments, satellite imagery [57], historical data [112,114], and manually collected data (physical quantification) [8,9]. Therefore, ensuring the quality of data from several sources is critical for applications using ML models. In some instances, the ML data acquisition process requires specialized skills and a huge cost of acquisition, such as data acquisition through hyperspectral imagining, making it difficult to acquire adequate data for use [73]. There is also the problem of the reliability of data due to inconsistencies in data collection methodologies [121]. Furthermore, the interaction between horticultural crops and their environment is influenced by weather, farming practices, the prevalence of pests and diseases, and soil composition. These factors vary from time to time and need continuous monitoring. Also, the interpretability of these parameters and knowing how they influence horticultural production require horticultural knowledge, which can pose a limitation to experts in other fields. There is also the problem of scalability in the application of ML in horticultural production. Small-scale trials in the application of ML techniques in horticultural production are usually easier, cheaper, and often show positive results. Scaling these results to larger horticultural operations may involve substantial cost, infrastructural requirements, and suitability to the existing technologies and operations.

Furthermore, another aspect of the limitations of the implementation of ML in horticultural production is the selection of appropriate performance criteria to evaluate model performance. While this review has covered performance metrics for classification tasks in ML, such as F1-score, precision, and recall (as discussed in Section 1.3), it is crucial to also consider some of the metrics used in regression tasks, which are foundational in several ML applications. The commonly used parameters to assess a model’s accuracy in regression tasks include R-squared (R^2^), the root mean squared error (RMSE), the mean absolute error (MAE), and the mean bias error (MBE). R^2^ is the coefficient of determination, which indicates the proportion of variance in the dependent variable that is predictable from the independent variables, providing insight into the explanatory power of the model [37,67]. However, according to Clark et al. [122], the R^2^ value does not give the overall picture of the performance of a model since it does not evaluate the bias in the predicted result. The RMSE and MAE measure the average magnitude of error between the predicted and actual values, with the RMSE being particularly sensitive to large errors [37,67], making it suitable for applications in tasks where such errors are unacceptable. The MBE assesses the average bias in predictions, helping identify systematic overestimations or underestimations by the model [123]. The relationship between RMSE, MAE, and MBE is expressed by the inequalities: MBE ≤ MAE ≤ RMSE ≤ √nMAE, where MSE and RMSE are preferred if the theoretical analysis on error measure is conducted as opposed to MAE because of the ease of applying analytical measures to MAE [123]. From the analysis of the metrics, the choice of evaluation criteria must vary based on the type of task and application in horticultural production. Therefore, the metrics should be carefully selected based on the data characteristics and the implications of different error types in the specific application context. For example, in financial forecasting for ML applications in retail, where outliers can disproportionately affect the model’s performance, MAE might be preferred due to its resistance to the influence of large errors. Analyzing these metrics provides a more comprehensive evaluation of model performance, ensuring that their applications are both robust and directly tailored to the specific challenges at each stage of horticultural production.

Having discussed the challenges and limitations associated with implementing ML techniques in horticultural production, possible future research directions could involve considering the integration of ML models with Internet of Things (IoT) devices such as sensors to enable real-time monitoring and control of horticultural environments, which would lead to higher efficiency. Also, there is a need to explore novel ensemble ML algorithms (since they consist of a combination of traditional ML models), to address the unique challenges of horticultural production. Finally, ML experts must collaborate with other horticultural value chain actors such as farmers, processors, transportation, and equipment manufacturers to ensure that the developed models are practical, effective, and aligned with industry needs.

## 7. Conclusions

Recent advancements in artificial intelligence, specifically machine learning, have significantly reduced manual labor in pre and postharvest activities, transforming the food value chain. The integration of machine learning into horticultural practices has not only revolutionized operations but also enhanced the speed and accuracy of various processes. This review has reported the current knowledge of ML models that predict and classify variables accurately as stand-alone models, such as the SVM, RF, KNN, DT, and LR, and showed that some models achieve better results when combined with other models (ensemble method). Based on the findings of the review, regression models such as LR, SVR, and RF are most promising for future research in prediction and forecasting because they allow prediction through time series and show the underlying relationship among variables. Furthermore, the capability of ensemble methods to boost ML models’ accuracy and reduce bias promises a great improvement in the adaptability of these models in postharvest loss quantification. Given the successful application of ML models in different horticultural practices, it could be a game changer for postharvest loss quantification in the near future. However, the application of the techniques on a commercial scale requires specialized skills and can be cost-intensive. As this review has shown, studies relating to the application of ML techniques in quantifying and predicting postharvest losses and waste of horticultural produce are lacking, hence highlighting the importance of this study. The current application of ML technology in horticultural production has been concentrated on pest and disease prediction, yield prediction, and the classification of fruit and vegetables in sorting and grading operations. The critical need for ML in quantifying postharvest losses and waste is evident, especially when considering its potential impact on policy formulation and implementation for food loss and waste reduction. Given these considerations, future research should leverage ML to quantify and predict postharvest losses and waste, enhancing data accuracy and facilitating timely interventions.

## Figures and Tables

**Table 1:** Summary of ML application in pest and disease prediction and detection.

Produce	Pest/Disease	Parameters Observed	Evaluation	Algorithms Applied	Results	Reference
Milk thistle	Smut fungus	Leaf spectra images	Discrimination between healthy milk thistle and those affected by smut fungus	SKN, CP-ANN, and XY-fusion	95.16 accuracy	[21]
Rice	Bakanae disease	Rice cultivars Tainan 11 and Toyonishiki seedlings; morphological and colour traits of healthy and infected rice seedlings	Detection of Bakanae disease in rice seedlings	SVM	87.9% accuracy	[52]
Papaya leaves	Abnormalities on papaya leaves	Leaf images	Identify between healthy and disease-infected papaya leaves	RF	70.14% accuracy	[50]
Multiple crops	Insect	Shape features extracted from the insect images	Classification and detection of insects in field crops	ANN, SVM, KNN, NB, and CNN	CNN provided the highest classification accuracy of 91.5% and 90% for 9 and 24 classes of insects	[51]
Wheat	Yellow rust	Leaf spectra images	Automatic detection of ‘yellow rust’ disease	ANN	99% accuracy	[55]
Rice	Brown planthopper	Weather and host plant phenology factors	Forecast the brown planthopper population	ANN, RF, and MLR	ANN: R^2^ = 0.770, RMSE = 1.686; RF: R^2^ = 0.754, RMSE = 1.737; and MLR model: R^2^ = 0.645, RMSE = 2.015	[49]
Crop leaf	Alternaria Alternate, Anthracnose, Bacterial Blight, and Cercospora leaf spot	Different leaf images	Identify between disease-infected and healthy leaves	SVM	Over 95% accuracy for disease-infected leaves and 98% accuracy for healthy leaves	[56]
Grape leaves	Black measles, black rot, and leaf blight	Leaf captured images	Diagnose and classify diseased-infected and healthy leaves	PCA and SVM	SVM classifier combined with linear kernel, using the GLCM features, produced a 98.71% accuracy	[53]
Date fruit	Date palm mite	Meteorological variables and physicochemical properties of date fruits	Prediction of date palm mite count on date fruits	LR and DFR	DFR performed better than LR in all the variables, with R^2^ of 0.842, 0.895, and 0.921 for MV, PPV, and MPPV, respectively. LR produced R^2^ of 0.464, 0.670, and 0.554 for MV, PPV, and MPPV, respectively.	[54]

Supervised Kohonen network (SKN); counter propagation artificial neural network (CP-ANN); support vector machine (SVM); random forest (RF); artificial neural networks (ANNs); K-nearest neighbors (KNN); naïve Bayes (NB); convolutional neural network (CNN); linear multiple regression (MLR); principal component analysis (PCA); gray-level co-occurrence matrix (GLCM); coefficient of determination (R^2^); root mean square error (RMSE); linear regression (LR); decision forest regression (DFR); meteorological variable (MV); physicochemical properties variables (PPVs); meteorological and physicochemical properties’ variables (MPPVs).

**Table 2:** Summary of ML applications in the prediction of crop loss due to natural causes.

Produce	Cause of Damage	Parameters Observed	Evaluation	Algorithms Applied	Results	Reference
Soybeans, wheat and corn	Hailstorm	Sentinel-1 and -2 images; data from damage evaluation	Detection of crop hailstorm damage	K-means clustering	87.01% accuracy.	[57]
Tea tree	Frost	Topography and meteorological data	Predict the occurrence of a tea-tree frost event; establish spatial distribution of frost damage to tea trees	SVM and ANN	SVM = 83.8% accuracy; ANN = 75% accuracy.	[59]
Wheat	Drought	Relative leaf area index, (RLAI), standardized precipitation index (SPI), and standardized soil moisture index (SSMI)	Drought risk assessment	MCWLA and RF and MCWLA and MLR	MCWLA and RF performed better with a RMSE = 6%, while MCWLA and MLR’s RMSE = 20%.	[58]
Rice, wheat, maize	Drought	Meteorological drought indices	Prediction of yield loss due to future drought	RF, GBM, and EML	EML (RF and GBM) outperformed other models with an RMSE = 0.390, 0.358, and 0.387 for rice, wheat, and maize, respectively.	[22]
Maize, wheat, sorghum, barley, teff	Drought	Meteorological and agricultural survey data	Prediction of crop loss due to drought	RF	81% accuracy.	[60]
Wheat	Lodging	UAS RGB images	Wheat lodging detection	RF, NN, and SVM	RF outperformed other models with an accuracy of 91%.	[61]
Multiple grass crops	Cold stress	Genomic features	Prediction of cold-responsive and non-responsive genes	RF	The model successfully predicted genes that would respond to cold stress in related plant species.	[62]

Support vector machine (SVM); artificial neural networks (ANNs); model to simulate the crop–weather relationship over a large area (MCWLA); random forest (RF); multiple linear regression (MLR); gradient boosting machine (GBM); ensemble machine learning (EML); neural network (NN); root mean square error (RMSE); unmanned aerial systems (UAS); red green blue (RGB).

**Table 3:** Summary of ML applications in yield prediction.

Produce	Parameters Observed	Evaluation	Algorithms Applied	Results	Reference
Coffee	Colour features in digital images	Automatic fruit count on coffee branches	SVM	Ripe–overripe: 82.54–87.83%; semi-ripe: 68.25–85.36%; unripe: 76.91–81.39% (visibility percentage of fruit).	[64]
Citrus fruit	Image features such as brightness and darkness	Identification of immature green citrus fruit	SVM	80.4% accuracy.	[65]
Agricultural yield	Historical agronomical, environmental, and economic data	Agriculture yield prediction	ENN and BPN	1.30 error rate.	[70]
Potatoes	Data on physicochemical properties of soil	Identification of variability in soil properties and potato yield	LR, EN, KNN, and SVR	SVR outperformed other models with an RMSE of 5.97, 4.62, 6.60, and 6.17 t/ha for all datasets, while KNN performed the poorest, with an RMSE of 6.93, 5.23, and 6.91 t/ha in three out of four datasets.	[66]
Irish potatoes and Maize	Historical harvest data and meteorological parameters	Variability in weather elements and Irish potatoes and maize yield	RF, PR, and SVR	RF outperformed other models with an RMSE of 510.8 and 129.9 for potato and maize, respectively, while SVR performed the poorest, with an RMSE of 971.6 and 212.4 for the same data set.	[37]
Multiple crops	Historical agronomical and environmental data	Yield prediction	LR, DT, EN, LR*, RR, PLSR, GBR, and LSTM	LSTM outperformed other models with an 86.3% accuracy, while PLSR performed the least with a 76.8% accuracy.	[36]
Soybean	Meteorological and historical yield data	Yield prediction	MLR, MLP, SVM, RF, XGBOOSTING, and GradBOOSTING	XGBOOSTING outperformed other models with an RMSE of 2.06 for calibration, while RF, XGBOOSTING, and GradBOOSTING performed better than other models for testing with an R^2^ of 0.71, 0.62, and 0.62, respectively.	[71]
Wild blueberry	Plant height, fruit production, slope, leaf loss, and blower damage	Mechanical harvesting yield loss	SVR, LR, and RF	LR outperformed other models with an R^2^ of 0.91, 0.87, 0.73, and 0.91 for Frank Webb, Tracadie, Cooper, and Small Scott, respectively. While SVR performed relatively well with an R^2^ of 0.93, 0.88, 0.79, and 0.07 for the same areas, respectively.	[66]
Wheat	Multi-source environmental variables such as satellite-based vegetation indices, climate data, and soil properties	Yield prediction	RF and SVM	RF with near-infrared reflectance of terrestrial vegetation (NIR_V_) and other covariates performed better in yield prediction with an R^2^ and an RMSE of 0.74 and 758 kg/ha, respectively, while SVM with the same variables produced an R^2^ of 0.69 and RMSE of 821 kg/ha.	[72]

Back propagation neural network (BPN); ensemble neural network (ENN); elastic net (EN); K-nearest neighbors (KNNs); support vector machine (SVM); support vector regression (SVR); polynomial regression (PR); linear regression (LR); random forest (RF); decision tree (DT), Lasso regression (LR)*; Ridge regression (RR); partial least square regression (PLSR); gradient boost regression (GBR); long short-term memory (LSTM); multiple linear regression (MLR); multi-layer perceptron (MLP); extreme gradient boosting (XGBOOSTING); gradient boosting (GradBOOSTING); coefficient of determination (R^2^); root mean square error (RMSE); near-infrared reflectance of terrestrial vegetation (NIR_V_).

**Table 4:** Summary of ML application in crop quality detection.

Produce	Parameters Observed	Evaluation	Algorithms Applied	Results	Reference
Cotton	Infrared hyperspectral transmittance images	Classification of foreign matter embedded inside cotton lint	SVM	Over 95% accuracy.	[74]
Papaya	Digital images	Prediction of the quality and ripening stages of papaya	LDA, QDA, LSVM, and QSVM	LDA and LSVM produced the highest result accuracy of 83.5% and 79.5%, respectively.	[75]
Wheat grains	Colour and texture features of wheat grain samples	Classification of wheat grain into ‘fresh’ and ‘rotten’	SVM, KNN, MLP, and NB	SVM produced the highest accuracy of 93% based on colour features, while the NB model produced the highest accuracy of 65% based on texture features.	[76]
Wheat seed	Shape, colour, and texture features	Identification and classification of seven-grain groups in wheat seed	LDA, QDA, LSVM, QSVM, and CSVM	QSVM produced the highest accuracy with 98.7, 98, 100, 97.3, 99.3, 99, 99.3, and 90.7% for sound white wheat, small white wheat, barley, rye, red wheat, broken white wheat, and shrunken white, respectively.	[81]
Avocados	Electromagnetic data from UHF RFID tags in contact with fruits	Automatic monitoring of avocado ripening	SVM	Over 85% accuracy.	[77]
Tomato	Colour features	Automatic classification of tomato ripeness stages	SVM and LDA	The one-against-one multi-class SVMs performed better than the one-against-all multi-class SVMs, and the LDA algorithms with 90.80, 84.80, and 84% accuracy, respectively.	[78]
Papaya	LBP, HOG, and GLCM features collected from image samples	Classification of maturity status of papaya fruits	KNN, SVM, and NB	Weighted KNN with HOG features performed better than other models with 100% accuracy and 0.0995 s training time.	[82]
Banana	Thermal images	Monitoring of fruit quality change	CNN	99% accuracy.	[83]
Loquat	Hyperspectral images	Classification of sound and defective loquat fruit	RF, XGBoost	XGBoost outperformed RF with 97.5, 96.7, and 95.9% accuracy for sound or defect; sound, internal, or external defect; and sound or purple spot, scar, bruising, or flesh browning, respectively.	[84]

Support vector machine (SVM); linear discriminant analysis (LDA); quadratic discriminant analysis (QDA); linear support vector machine (LSVM); quadratic support vector machine (QSVM); K-nearest neighbor (KNN); multi-layer perceptron (MLP); naïve Bayes (NB); linear discriminate analysis (LDA); quadratic discriminate analysis (QDA); quantized support vector machine (QSVM); cubic support vector machine (CSVM); ultra-high frequency (UHF); radio frequency identification (RFID); local binary pattern (LBP); histogram of oriented gradients (HOG); gray level co-occurrence matrix (GLCM); convolutional neural networks (CNNs), random forest (RF); extreme gradient boost (XGBoost).

**Table 5:** Summary of ML applications in fruit and vegetable sorting/grading.

Produce	Parameters Observed	Evaluation	Algorithms Applied	Results	Reference
Coconut	Acoustic signal	Classification of coconut fruit into pre-mature, mature, and over-mature	ANN, RF, and SVM	ANN: train = 79.32%; test = 81.74%; RF: train = 90.98%; test = 83.48%; SVM: train 88.35%; test = 80.00%.	[23]
Vegetable oils	Fatty acids profile	Discrimination of premium quality oil from inexpensive edible oils	RF	Cis-monounsaturated fatty acids in tea oil (79.48%) were close to the expensive extra virgin olive oil (80.71%) and could be a substitute.	[91]
Banana	Colour and size features	Classification of bananas into extra class, class I, class II, and reject class	ANN, SVM, and RF	RF provided the highest classification accuracy of 94.2%. Without the reject class, at least 97% accuracy was achieved in the other classes.	[87]
Tomatoes	Colour image processing	Detection of defects in cherry and heirloom tomatoes	SVM models, ANN, and RF	RBF-SVM performed better than other models, with an accuracy of 0.9709 for the healthy and defective tomatoes category.	[85]
Multiple fruits and vegetables	Colour, texture and geometrical features	Detection of type and grading of fruits and vegetables	LR, SRC, ANN, and SVM	SVM outperformed other models with 97.63% and 96.59% accuracy for the detection of the type of vegetable or fruit and grading of vegetable and fruit, respectively.	[92]
Apples and mangoes	Digital images of fruits	Classification of fruits into damaged or good fruit	KNN, SVM, and C4.5	SVM outperformed other models with a 98% accuracy.	[93]
Hawthorns	Colour and texture features	Classification of fruits into unripe, ripe, and overripe	ANN and SVM	ANN performed better than SVM with 99.57, 99.16, and 98.16% accuracy for training, validation, and testing respectively.	[94]
Bell pepper	Colour, texture and size features	Prediction of maturity stage and size of bell peppers	ANN and MLP	MLP classifier performed better with 93.2%, 86.4%, 84%, and 95.7% for accuracy, precision, sensitivity, and specificity, respectively.	[95]
Apple	Colour features	Automatic inspection and classification of apple fruit	SVM, KNN, XGBoost, and CatBoost	SVM outperformed other models by classifying the three types of apple samples with an accuracy of 96.7%.	[79]
Parijoto Fruits	Texture features	Classification of parijoto fruits into “good”, “rotten”, and “defects”	KNN	80% accuracy.	[96]

Artificial neural networks (ANNs); random forest (RF); support vector machine (SVM); linear regression (LR); sparse representative classifier (SRC); multi-layer perceptron (MLP); K-nearest neighbor (KNN); extreme gradient boosting (XGBoost); categorical boosting (CatBoost).

**Table 6:** Summary of ML application in crop detection and cultivar classification.

Produce	Parameters Observed	Evaluation	Algorithms Applied	Results	Reference
Korla fragrant pear	Hyperspectral images of pear fruit	Differentiating Korla fragrant pears into the deciduous–calyx or persistent–calyx categories.	SPA and SVM	SPA: 93.3% accuracy; SVM: 96.7% accuracy.	[98]
Rice	Sentinel-1 images	Infield rice crop detection.	SVM, RF, KNN, and normal Bayes (NB)*	Accuracy and kappa values for all models are greater than 97% in all metrics.	[97]
Apricots	Shape features	Classification of apricot cultivars.	DT, KNN, naïve Bayes (NB), LDA, SVM, and BPNN	SVM integrated with SPA has the highest accuracy, with 90.7%.	[99]
Wheat	Physical features	Classification of wheat seeds into 3 varieties.	KNN, NB, CART, and EM	EM outperformed other models with an accuracy of 95%.	[101]
Wheat	DSIFT features	Classification of wheat seeds into 40 varieties.	SVM	88.33% accuracy.	[102]
White mustard seeds	Texture features	Classification of traditional and double-low cultivars.	Multiple classifiers	R channel produced the highest accuracy with 93%, and 83% accuracy was achieved in RGB colour space when compared to other channels and colour spaces.	[103]
Corn seed	Digital image	Classification of 6 varieties of corn seeds.	RF, BN, LB, and MLP	MLP outperformed other models with a 98.83% accuracy.	[104]
Multiple seeds	Digital image	Classification of 14 different seeds.	CNN, KNN, DT, NB, RF, AdaBoost, and LR	CNN achieved 99% accuracy in comparison with other models.	[105]
Dry beans	Dimensional and shape features	Classification of 7 different varieties of dry beans.	MLP, SVM, KNN, and DT	Overall, SVM outperformed other models with an accuracy of 93.13% and classified the individual varieties—Barbunya, Bombay, Cali, Dermason, Horoz, Seker, and Sira—with 92.36%, 100.00%, 95.03%, 94.36%, 94.92%, 94.67%, and 86.84% accuracy, respectively.	[106]
Pineapple	Thermal image features	Classification of pineapple into 3 different cultivars.	LDA, QDA, SVM, KNN, DT, and NB	SVM achieved 100% accuracy in comparison with other models.	[39]
Barley	Satellite NDVI and Finnish Food Authority reference data	Classify field parcels with and without crop loss.	LR, DT, RF, and MLP	RF and mean and MI (recommended). Classification of loss: within a year is possible. Between years is difficult.	[44]
Multiple crops	Spectral and textural features	Classification of crops into herbaceous crops or woody crops.	C4.5 DT, LR, SVM, and MLP	MLP and SVM achieved the highest classification accuracy of 88% each as single classifiers, while SVM and SVM performed best among the hierarchical classifiers by improving accuracy to 89%.	[107]

Successive projections algorithm (SPA); support vector machine (SVM); random forest (RF); K-nearest neighbors (KNNs); normal Bayes (NB)*; decision tree (DT); naïve Bayes (NB); linear discriminant analysis (LDA); back propagation neural network (BPNN); classification and regression tree (CART); ensemble methods (EMs); dense scale-invariant feature transform (DSIFT); BayesNet (BN); LogitBoost (LB); multi-layer perceptron (MLP); convolution neural network (CNN); logistic regression (LR); quadratic discriminant analysis (QDA); normalized difference vegetation index (NDVI).

**Table 7:** Summary of ML application during retail.

Produce/Variable	Parameters Observed	Evaluation	Algorithms Applied	Results	Reference
Palm oil	MEODA data	Prediction of price	RF	91.11% accuracy.	[109]
Consumer behaviour	Kaggle repository	Prediction of consumer behaviour	RF	94% accuracy.	[110]
Sales	Daily sales data	Prediction of product and store sales	XGBoost, ARIMA, and LSTM	XGBoost performed better in comparison with other models with an RMSE of 0.878, while ARIMA and LSTM achieved 1.092 and 0.924, respectively.	[111]
Tomato, potato and onion	Daily sales data	Demand forecast of vegetables	LSTM, RFR, GBR, XGBoost, SVR, and ARIMA	LSTM and SVR outperformed other models. LSTM = RMSE values ranged between 3.75 and 15.68, 7.03 and 21.6, and 8.20 and 20.77 for tomato, potato, and onion, respectively. SVR = RMSE values ranged between 6.28 and 21.11, 14.04 and 28.88, and 7.92 and 26.8 for tomato, potato, and onion, respectively.	[113]
Sales	Historical sales data	Sales forecasting	LR, RR, and XGBoost	XGBoost performed better in comparison with other models with an RMSE of 0.655, while LR and RR achieved 0.783 and 0.774, respectively.	[112]
Perishable produces	Historical data	Demand forecast of perishable produces	SVM	MAPE = 0.869.	[114]
Onion and potato	Daily sales data	Daily demand forecast	ARIMA	MAPE is 28.296 for onion and 29.51 for potato.	[115]
Banana	Daily sales data	Sales forecasting	Seasonal naïve forecasting, SARIMA, MLPNN-1, MLPNN-2, SARIMA-MLR, and SARIMA-QR	SARIMA-MLR and SARIMA-QR both performed better than other models with an RMSE of 19.14 and 19.35, respectively.	[116]

Random forest (RF); eXtreme gradient boosting (XGBoost); autoregressive integrated moving average (ARIMA); long short-term memory (LSTM); random forest regression (RFR); gradient boosted regression (GBR); support vector regression (SVR); root mean square error (RMSE); Ridge regression (RR); support vector machine (SVM); mean absolute percentage error (MAPE); seasonal autoregressive integrated moving average (SARIMA); multi-layered perceptron neural network (MLPNN); multiple linear regression (MLR); quantile regression (QR).

## Data Availability

All data are made available in the manuscript.
